# Health-Related Quality of Life in End-Stage Renal Disease Patients and Healthy Individuals

**DOI:** 10.31661/gmj.v9i0.1987

**Published:** 2020-12-29

**Authors:** Niyan Hakeem Ismael, Aso Omer Rashid

**Affiliations:** ^1^Faculty of Medical Sciences, College of Nursing, University of Sulaimani, Urological Department. Sulaymaniyah, Iraq; ^2^Faculty of Medical Sciences, College of Medicine, University of Sulaimani Urological Department, Sulaymaniyah, Iraq

**Keywords:** End-Stage Renal Disease, Health-Related Quality of Life, Kidney Transplantation, Chronic Disease

## Abstract

**Background:**

Health-related quality of life (HRQOL) is an important outcome measure in patients with end-stage renal disease (ESRD). HRQOL is assumed to improve with kidney transplantation and compared to hemodialysis. However, there is no evidence regarding HRQOL to support the optimal treatment choice for patients on hemodialysis who hesitate opting for transplantation. Therefore, this study aims to compare HRQOL between patients with ESRD and healthy individuals.

**Materials and Methods:**

This case-control study was performed of 50 patients with ESRD under hemodialysis and 100 healthy participants as controls. HRQOL was assess using the SF-36 questionnaire. Data was analysis by using linear regression to compared HRQOL between groups, and adjusted for age, gender, dialysis duration.

**Results:**

Most of the patients were males (62%) and aged 21 to 60 years old (82%). The patients and healthy subjects were significantly different in terms of the presence of chronic diseases (P<0.05). ESRD patients had a significantly lower level of satisfaction with health and function, family and friends, and social and psychological functions. The patients’ quality of life was not significantly affected by their demographic characteristics, including age, gender, educational level, marital status, and financial status. However, there was a significant association between chronic disease and HRQOL among ESRD (P=0.0001).

**Conclusion:**

ESRD has a remarkably negative effect on the patients’ quality of life and satisfaction with important domains of life. HRQOL among patients with end-stage renal disease can be affected by the associated chronic diseases.

## Introduction

Health-related quality of life (HRQOL) is an important indicator of well-being in patients with end-stage renal disease (ESRD) and is associated with survival and clinical outcomes [1–4]. Compared to the general population, patients with ESRD have severely diminished HRQOL, by some needed even lower than in diseases such as congestive heart failure, chronic lung disease and/or cancer [5].

The preferred treatment for ESRD is kidney transplantation, which is associated with improved HRQOL and survival [6]. However, because of the limited availability of donor kidneys and because of transplant failure, many patients have to remain on dialysis. An alternative to conventional dialysis modalities is frequent nocturnal hemodialysis. With this treatment, patients dialyze almost daily and twice as long (7–8 hours), generally at home. Thus, this treatment removes fluid more slowly and clears more solutes such as urea and phosphate [7]. Worldwide, it is estimated about 200 million people have chronic kidney disease (CKD), and correspondence to the burden of CKD continues to increase in low- to middle-level income countries globally [8]. The annual mortality rate of CKD was 14.8%–15.7% in the United States between 2006 and 2008 [9]. While ESRD, which is more devastating medical, social, and economic problem, needs more supervision and medical cares. It is associated with1-year mortality of 23.5% in the USA with the cardiac causes consist of 50% of all deaths [10,11] Globally, more than one million people die annually from ESRD [8]. Instead of traditional endpoints assessment for the effect of interventions on patients, the quality of life (QoL) measures have become increasingly used in the recent decades with changing the pattern of illness in developed and developing countries [12,13]. Traditional morality based measures present information about the lowest levels of health only. However, they reveal little about the critical aspects of health and well-being [14]. On the other hand, the different domains of HRQOL that measure the state of well-being, including the perception of general health in three dimensions of physical, psychological, and social states that is primary interested as an indicator of the efficacy of receiving therapeutic cares [15]. In the management of patients of ESRD, although the progress has been achieved in therapeutic agents. Using dialysis process in passing off superfluous, the longevity of patients has been increased, but several metabolic complications threaten the HRQOL. Thus, the limitations would arise in different aspects of daily activities. Many of these patients are unable to cope with possible limitations; therefore: the psychological along with physical and social problems could affect their health states [16,17].

## Materials and Methods

 This case-control study was performed of 50 patients with ESRD under hemodialysis and 100 healthy participants as controls. The patients were recruited from the dialysis center of Shar Hospital in Sulaimani city. The inclusion criteria were the ESRD with at least three months duration of dialysis and aged 20–80 years. The controls were selected from healthy participants of patients’ visitors or outpatients clinic. In control selection, participants with debilitating conditions such as diabetes, renal failure, history, cardiovascular disease, heart surgery, cancers, dementia, overt disability, and congenital disorders were excluded. Additionally, those who lost their close relatives within the previous six months were also excluded from control selection. For each case, two gender and age-matched controls were selected from patients’ visitors or outpatient clinics of the hospital. The demographic data were collected using face to face interviews conducted, and SF-36, standard questionnaire of HRQOL, was used. The validity and reliability of this questionnaire were assessed in several reports. This questionnaire included 36 items that assess the HRQOL in five dimensions. In each dimension, the score of items was transformed as a subscale from 1 (worse health) to 6 (best health). Also, the internal consistency of items within each subscale was evaluated by the reliability coefficient for rating data. The duration of dialysis and demographic data such as age, gender, marital status, educational level, and residence area were collected.

###  Statistical Analysis

 Statistical analysis was used by Statistical Program for Social Sciences (SPSS) software version 21 (IBM Corp. Released 2013, Armonk, New York, USA) and data presented as mean ± standard deviation (SD). The overall QoL as weighted QoL of specific subscales were calculated according to case status, gender, age group, educational level. Also, Mann–Whitney and Kruskal–Wallis tests were used to avoid any distributional assumption in comparison of QoL between cases and healthy participants. In addition, the multiple linear regression models were applied to adjust the effect of possible potential confounding factors such as age, gender, educational level, marital status, residence area, and various dimensions of QoL. The adjusted regression coefficients of multiple linear regressions show the adjusted mean difference between groups of binary predictor factors that entered into the model. The P<0.05 was considered statistically significant.

## Results

 The mean age was 41.03±15.004 years, and most of the participants in both case and control groups belonged to the age groups 21-40 and 41-60 years (82% of the cases and 86% of the controls). Regarding their gender, most of the case (62%) and control (60%) were males. In terms of their education level, almost equal percentages of the cases and controls were illiterate (28% vs. 22%) or finished primary school (28% vs. 25%), secondary school (26% vs. 30%), and institute or college (18% vs. 23%). Regarding their marital status, most of the case (66%) and control (72%) groups were married. With regard to their financial status, most of the cases (62%) and controls (61%) had barely sufficient financial status. The cases and control were not significantly different in terms of the abovementioned demographic characteristics; therefore, they were homogenous in terms of their demographics. With regard to having chronic diseases, there was a significant difference between the cases and controls (P=0.0001), such that most of the cases (88%) had chronic disease, while more than half of the controls (57%) did not (Table-1). Comparing the cases and controls regarding different important domains and satisfaction with them, the results showed that the two groups were significantly different in terms of their health and functioning, family and friends, social life, and psychological/spiritual state and their satisfaction with such domains (P=0.0001). These results demonstrated that the level of satisfaction with these domains was significantly higher in the controls compared to the cases (Table-2). The control participants were remarkably more satisfied than the ESRD patients with their health and functioning (44% vs. 66%), family (43% vs. 69%), social and economic status (45% vs. 63%), and psychological and spiritual functioning (52% vs. 70%). Moreover, the overall satisfaction in the case and the control group was 46% and 67%, respectively. Data showed means scores of QoL domains of two groups were significantly different regarding health and functioning, family, social functioning, and psychological functioning (P=0.0001, Table-3). Regarding the relationship between the patients’ demographic characteristics and their overall QoL, the results showed that there was not significantly correlated with their age (P=0.437), gender (P=0.84), educational level (P=0.16), marital status (P=0.91), and financial status (P=0.18). However, a highly significant correlation was observed between their overall QoL and the presence of chronic diseases (P=0.0001, Table-4).

## Discussion

Patients with ESRD are usually at a high risk of poor survival and adverse clinical outcomes, causing their HRQOL to undergo a remarkable decrease [3-5]. In this regard, the present study was carried out in order to compare the demographic characteristics and different domains of QoL in patients with ESRD to take into account those parameters and come up with a higher QoL among such patients. The results demonstrated that most of the ESRD patients aged between 21 and 60 years. Almost in line with this finding, Hochman *et al*. studied prevalence and incidence of ESRD in patients ≥ 18 years, reported that ESRD was more prevalent among individuals over the age of 45 years compared to those below this age [19]. Moreover, as shown by the results, ESRD was more prevalent among male patients. In line with this finding, Stats *et al*. pointed out that men were more likely (64%) to develop ESRD than women [20]. As revealed by the results of the current study, the patients with ESRD were not significantly different in terms of parameters such as age, gender, education level, marital status, and financial status. Therefore, none of these can be considered as significantly decisive risk factors for ESRD; however, it was observed that a larger number of male and older age individuals developed ESRD, such that ESRD was observed in 62% of males, while 38% of the females had it. Also, individuals aged between 21-60 years were more afflicted by the disease than younger ones. In line with our study, other studies have shown that older age and male sex are risk factors for ESRD [21-23]. Our results indicated that patients with ESRD were significantly different from healthy individuals in terms of having the chronic disease. This finding is in good agreement with the results of the studies carried out by Wu *et al*. (2018) and Narres *et al*. (2016) that pointed out that there were a significant association between the presence of ESRD and developing diabetes [24,25]. Similarly, Alalawi *et al*. have also reported that 57% and 12.4% of the ESRD cases resulted from diabetic nephropathy and hypertension, respectively [26]. It has also been reported that anxiety and depression are associated with ESRD [27]. The healthy subjects were compared with the patients with ESRD in terms of their important domains of life and their satisfaction with them. These domains included health and functioning, family and friends, social functioning, and psychological and spiritual functioning. The results revealed that the two groups were significantly different in all these domains. In other words, it was observed that the ESRD patients had remarkably lower levels of satisfaction with these important domains of their lives, indicating that they had a lower level of QoL significantly. In line with these findings, Kutner *et al*. reported that patients with ESRD have a high level of functional impairment [28]. Also, Gerogianni *et al*. (2016) stated that ESRD patients who had undergone hemodialysis were less satisfied with their relationships with their family and friends, such that they felt they were a burden to them [29]. The results regarding the low satisfaction of the patients with their social functioning and mental/spiritual functioning have been supported by the study carried out by Rostami *et al*. that reported a poor level of social and mental functioning in ESRD patients undergoing hemodialysis [30]. In this regard, studies have suggested that social and familial support can raise the overall quality of life in patients with ESRD [31,32]. The relationship between the ESRD patients’ demographics and their overall QoL was compared, and the results showed that none of the demographic characteristics (i.e., age, gender, educational level, marital status, and financial status) was significantly associated with their overall QoL. Despite insignificant relationship between overall QoL and age, it was noticed that the ESRD patients’ overall QoL dropped with increasing age, such that patients under the age of 20 years had the highest overall QoL, while those aged over 60 years had the lowest. In line with this result, Cruz *et al*. concluded that QoL was remarkably lower in older ESRD patients, particularly regarding their physical functioning [33]. The patients’ gender has no significant effect on their overall QoL. In this regard, the literature has revealed contradictory results. For example, Rostami *et al*. stated that QoL was better in men than women [30], while Bayoumi *et al*. reported that women had a higher level of QoL than men [34]. However, Peng *et al*. have claimed that since women undergo deeper psychological disorders as a result of ESRD, they have a lower level of overall QoL [35]. In line with the results of the present study, Zhou *et al*. reported that factors like financial status, marital status, and dialysis methods have no significant effect on scores of QoL [36]. According to the results of the present study, the chronic diseases associated with ESRD had a significant effect on the patients’ overall QoL. Chronic diseases, no matter what other diseases they are associated with, have been reported to have a remarkable effect on the patients’ quality of life. This finding is in line with the results of the study carried out by Megari. reported confirmed that chronic diseases remarkably affect the patients’ QoL; therefore, nurses and social workers are highly recommended to provide such patients with sufficient support in order to enhance their HRQOL [37]. Similar findings have also been reported by Pengpid and Peltzer [38].

## Conclusion

 ESRD can affect both genders and all age groups; however, it is more prevalent among males and older-age patients. HRQOL among ESRD patients can be negatively affected by both the disease itself and others associated with chronic diseases. ESRD patients have a low level of satisfaction with health and functioning, family and friends, social functioning, and psychological functioning. QoL in ESRD patients was not significantly affected by their demographic characteristics.

## Conflict of Interest

 Authors declare there was no conflict of interest.

**Table 1:** Socio-Demographic Data of Participants

**Characteristics**	**Case group**		**Control group**		**P-value**
	**n**	**%**	**n**	**%**	
**Age group, y**					
≤ 20	5	10	4	4	0.366
21 - 40	20	40	50	50	
41 - 60	21	42	36	36	
>60	4	8	10	10	
**Gender**					
Female	19	38	40	40	0.813
Male	31	62	60	60	
**Education levels**					
Illiterate	14	28	22	22	0.755
Primary school	14	28	25	25	
Secondary school	13	26	30	30	
Institute or college	9	18	23	23	
**Marital status**					
Single	13	26	21	21	0.747
Married	33	66	72	72	
Widowed/separated	4	8	7	7	
**Financial status**					
Sufficient	2	4	4	4	0.993
Barely sufficient	31	62	61	61	
Insufficient	17	34	35	35	
**Chronic disease**					
No	6	12	57	57	0.0001
Yes	44	88	43	43	

**Table 2:** Distribution of the Mean Scores of Participants’ Satisfaction and Important Domains

**HRQPL**	**Case group**		**Control group**		**P-value**
	**Mean**	**SD**	**Mean**	**SD**	
**Satisfactions domains**					
Health and functioning	44.40	10.12	65.35	6.11	0.0001
Family and friend	44.40	14.97	70.27	8.47	0.0001
Social	45.92	11.02	62.04	6.79	0.0001
Psychological / spiritual	53.71	8.54	71.00	5.39	0.0001
Total important	47.11	9.41	67.16	5.05	0.0001
**Important domains**					
Health and functioning	43.83	9.55	66.00	4.99	0.0001
Family and friend	42.27	12.57	66.73	7.99	0.0001
Social	45.17	9.78	63.38	9.78	0.0001
Psychological / spiritual	50.33	8.09	69.090	6.01	0.0001
Total important	45.40	8.75	66.50	4.54	0.0001

**Table 3:** Distribution of Differences in Mean Scores of QoL Domains in Case and Control Groups

**Variables**	**Mean **	**Std. Error **	**95% CI **		**P-value**
			**Lower**	**Upper**	
**Health functioning**	-21.22	1.155	-23.50	-18.94	0.0001
**Family**	-25.167	1.517	-28.17	-22.17	0.0001
**Social **	-18.10	1.271	-20.62	-15.59	0.0001
**QoL psychological**	-18.43	0.975	-20.36	-16.50	0.0001
**QoL overall**	-20.73	1.031	-22.77	-18.69	0.0001

**Table 4:** Distribution of Differences in Mean Scores of QoL According to Patients’ Characteristics

**Characteristics**	**Overall QoL** **(Mean ± SD)**	**P-value**
**Age group, y**		
≤ 20	51.0 ± 9.6	0.437
21- 40	44.6 ± 9.3	
41- 60	46.8 ± 8.1	
>60	43.8 ± 1.9	
**Gender**		
Female	45.8 ± 6.3	0.84
Male	46.3 ± 9.7	
**Educational level**		
Illiterate	41.9 ± 3.8	0.16
Primary school	46.9 ± 9.9	
Secondary school	47.7 ± 6.9	
Institute or college	49.1 ± 11.8	
**Marital status**		
Single	46.7 ± 12.5	0.91
Married	46.1 ± 7.1	
Divorced	44.6 ± 2.3	
**Financial status**		
Sufficient	44.5 ± 2.7	0.18
Barely sufficient	47.8 ± 9.8	
Insufficient	43.2 ± 4.8	
**Chronic disease**		
No	61.9 ± 11.9	0.0001
Yes	43.9 ± 5.1	
